# A Case of Surgery Cancellation Following the Discovery of Anisocoria After Induction of General Anesthesia

**DOI:** 10.7759/cureus.33803

**Published:** 2023-01-15

**Authors:** Erika Harada, Aiji Sato-Boku, Mayuko Kanazawa, Naoko Tachi, Masahiro Okuda

**Affiliations:** 1 Department of Anesthesiology, Aichi Gakuin University School of Dentistry, Nagoya, JPN

**Keywords:** cerebrovascular disorder, general anesthesia, adie’s pupil, pupillotonia, anisocoria

## Abstract

Anisocoria after induction of general anesthesia may indicate a severe cerebrovascular disorder. We encountered a case in which anisocoria in the left and right eyes was noticed after induction of general anesthesia, and the surgery was canceled. The patient was a 53-year-old woman with a history of hypertension. She received general anesthesia 10 years ago, but the details were unknown.

Anesthesia was induced with propofol, remifentanil, and rocuronium, followed by nasal intubation. No significant change was observed in vital signs during the induction of anesthesia. After intubation, the pupils were checked according to the protocol for observing pupil diameter. The pupil size was found to be unequal, measuring 1 mm in the left eye and 4 mm in the right eye. A cerebrovascular disorder was suspected; thus, the surgery was canceled, and the patient was awakened and extubated. Neurological symptoms such as limb movements were checked after awakening, and no disorientation or motor dysfunction was detected in the patient. However, her pupils remained unequal, measuring 2 mm in the left eye and 4 mm in the right eye. Regarding light reflex, the left eye was miotic, but the right eye remained mydriatic. The pupillary symptom persisted even during discharge the next day. Since our hospital is a solely dental hospital, following discharge, after consulting the ophthalmology department of a nearby medical university hospital, the patient was diagnosed with pupillotonia, as she had been experiencing light dazzling in only her right eye for seven years, had no light reflex but near reflex, and was miotic due to the use of pilocarpine hydrochloride eye drops, which promotes miosis.

The patient has had these symptoms in the right eye for seven years, and it is possible that she had anisocoria during the preoperative examination at this time. If anisocoria had been detected and examined carefully during the preoperative examination, there would have been no need to cancel the surgery. In this case, we strongly felt that the pupils must be checked during the preoperative examination.

## Introduction

Anisocoria is defined as pupils with a diameter difference of >0.4 mm between the left and right eye [1]. As the appearance of anisocoria after induction of general anesthesia may indicate severe cerebrovascular disorder [2], immediate action is required. This is because hemodynamics may fluctuate significantly when general anesthesia is introduced. The history of head trauma or Adie syndrome is well-known as the main cause of anisocoria during general anesthesia. Nevertheless, anesthesiologists tend to forget to inquire about the history of head trauma or Adie syndrome at preoperative examination and forget to check the pupils; thus, the cause is often overlooked.

We herein report a case in which anisocoria was noticed after induction of general anesthesia, following which surgery was canceled. We obtained the patient’s written consent for this case report. In addition, we sought ethical considerations, such as personal information protection.

## Case presentation

The patient was a 53-year-old woman with a body height of 150 cm and a weight of 55 kg. She was scheduled for cyst extraction and tooth extraction for a jaw cyst of the right mandibular third molar. She had a history of hypertension and was taking calcium channel blockers (amlodipine besylate 2.5 mg/day) orally. Preoperative examination revealed no abnormalities. Although we should have checked the pupillary system during the examination according to the preoperative protocol, the diameter of the left and right pupils was not checked in this patient. The patient received general anesthesia for tonsillectomy 10 years ago, but the details were unknown.

**Figure 1:**
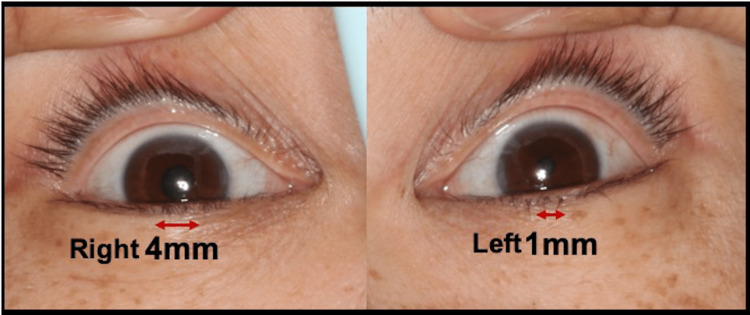
Pupil diameter after tracheal intubation

Premedication was not administered. The blood pressure (BP) at admission was 168/102 mmHg. After securing the intravenous access, the patient was anesthetized with 60 mg propofol and 0.3 μg/kg/min remifentanil. After administering 35 mg rocuronium, tramazoline hydrochloride using a cotton swab was applied to the left and right nasal cavities, and nasotracheal intubation through the right nasal cavity was performed. Intubation was performed smoothly in a short period. BP immediately after tracheal intubation was 153/94 mmHg. After intubation, the diameters of the left and right pupils were evaluated and found to be unequal, measuring 1 mm in the left eye and 4 mm in the right eye (Figure 1).

A cerebrovascular disorder was suspected; thus, the surgery was canceled to check the neurological symptom, and the patient was awakened and extubated. Neurological symptoms were evaluated after awakening, and the patient was found to have no disorientation or motor dysfunction. However, her pupil diameter did not improve and was unequal (2 and 4 mm in the left and right eyes, respectively). Regarding light reflex, the left eye was miotic, but the right eye remained mydriatic. The pupillary sign persisted even during discharge the next day.

After discharge, the attending physician referred the patient to the neurology department of a nearby medical university hospital for consultation. One week after the first visit to the neurology department, head and neck magnetic resonance imaging and chest computed tomography revealed no abnormal findings. Subsequently, the patient was diagnosed with pupillotonia after consulting the ophthalmology department of the same university hospital as she had been experiencing light dazzling in only her right eye for seven years, had no light reflex but near reflex, and was miotic due to the use of pilocarpine hydrochloride eye drops, which promotes miosis.

## Discussion

Pupillary muscles include sphincter pupillae muscles that constrict the pupil and dilator pupillae muscles that dilate the pupil; sphincter pupillae muscles are controlled by parasympathetic nerves (oculomotor nerves), whereas dilator pupillae muscles are controlled by sympathetic nerves. In the cerebrovascular disorder suspected in our case, anisocoria appeared because oculomotor nerves were compressed and blocked. In addition, in pupillotonia diagnosed in this case, it is thought that postganglionic fibrotic disorder of oculomotor nerves causes anisocoria, and this pupillotonia is called Adie’s pupil [1]. When accompanied by the disappearance or weakening of the Achilles tendon reflex and knee jerk reflex, it is called Adie syndrome, and it is often noticed following the occurrence of anisocoria during general anesthesia [3-6]. Regarding other causes of anisocoria, it has been reported that it occurred in one patient after the patient touched one eye with the finger that had been used to touch the scopolamine patch applied to the auricle for postoperative nausea and vomiting (PONV) prevention before surgery [7]. It has also been reported that anisocoria occurred when one eye was exposed to drugs after phenylephrine and lidocaine solution was sprayed for fiberoptic intubation before surgery [8]. In addition, it has been reported that anisocoria occurred after phenylephrine and lidocaine solution was administered to the nasal cavity for nasotracheal intubation refluxed via the nasolacrimal duct and infiltrated one eye [9]. In all of the above reports, anisocoria was noticed after the induction of general anesthesia, but surgery cancellation was not required as the cause was known. In addition to drugs, it has been reported that anisocoria was noticed after the induction of anesthesia. In that case, due to motor neuropathy of one eye, parasympathetic stimulation of anesthetic induction drugs was attenuated [10]. It has also been reported that despite tonic pupils due to previous facial trauma, anisocoria was not discovered before surgery but after induction of anesthesia [11]. Cerebrovascular disorders were suspected in all these cases, and the surgery was canceled.

Anisocoria is observed in about 10-20% of healthy adults, and there are various cases, from physiological cases and cases induced by drugs to cases requiring emergency treatment due to unknown causes [12]. If anisocoria is detected preoperatively or the patient complains of such symptoms, we should pause and ask to see an ophthalmologist. If anisocoria does not require intervention, the patient may proceed with the scheduled surgery because anisocoria is a preoperative finding that does not require intervention.

In this case, we discovered anisocoria after induction of general anesthesia, but the patient has had the symptom in the right eye for seven years; thus, there could have been anisocoria during the preoperative examination. If anisocoria had been detected preoperatively and examined carefully, there would have been no need to cancel the surgery. In this case, we strongly felt the need to check the pupils and examine them in more detail during the preoperative examination.

## Conclusions

We encountered a case in which anisocoria in the left and right eyes was noticed after induction of general anesthesia, and the surgery was canceled. In this case, the diameters of the left and right pupils were unequal; thus, a cerebrovascular disorder was suspected, and the patient was awakened. The prolonged surgery was performed after a close examination of anisocoria.

When the cause of anisocoria is unknown, it is an emergency, except when the cause of anisocoria is obvious preoperatively or when it is thought to be drug-induced. In our case, because it was highly likely that the patient had anisocoria before, checking it using a pen light during the preoperative examination was necessary.
